# Electropolymerized 1D Growth Coordination Polymer for Hybrid Electrochromic Aqueous Zinc Battery

**DOI:** 10.1002/advs.202101944

**Published:** 2021-09-17

**Authors:** Wei Church Poh, Xuefei Gong, Fei Yu, Pooi See Lee

**Affiliations:** ^1^ School of Materials Science and Engineering Nanyang Technological University Singapore 639798 Singapore; ^2^ Singapore‐HUJ Alliance for Research and Enterprise (SHARE) Nanomaterials for Energy and Water Nexus (NEW) Campus for Research Excellence and Technological Enterprise (CREATE) 1 Create Way Singapore 138602 Singapore

**Keywords:** aqueous electrolyte, coordination polymer, electrochromism, electropolymerization, 1D, zinc batteries

## Abstract

Organic materials are always viewed as promising electrochromic (EC) materials due to their synthetic versatility, color tunability, ready processability, and derivability from sustainable feedstocks. Most organic materials, however, are prone to undesirable redox side reactions in the presence of oxygen and water. As such, redox–active organic layers are often used in tandem with organic electrolytes to preserve their electrochemical stability. With the growing interest in electronics that are environmentally sustainable and biologically safe, developing aqueous‐compatible organic materials is gaining growing interest. Herein, a rationally designed iron terpyridyl coordination polymer (CP) is prepared by controlled electropolymerization for realization of aqueous compatible EC and energy storage applications. Detailed analysis is established, showing that the CP grows in a 1D fashion and exhibits a predominant capacitive behavior which is reflected from its rapid charge–transfer kinetics. Taking this as an advantage, an integrated hybrid electrochromic zinc battery device is demonstrated with high color contrast, fast response time, and good endurance.

## Graphical abstract

Electropolymerization of rationally designed iron–terpyridyl complexes forms 1D growth coordination polymer with the ability to electrochemically switch between the initial purplish‐blue state and the bleached transparent state in an aqueous electrolyte. The modified electrode is used to construct an integrated hybrid electrochromic zinc battery exhibiting both electrochromic and energy‐storage abilities with good endurance in the aqueous environment.

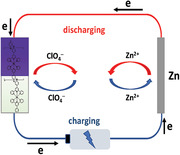

## 1 Introduction

With the advent of smart technology, innovative integration of several advanced functionalities into one electronic device is highly sought‐after and has received tremendous attention in research, for instance, energy‐storage systems have been made self‐powering,^[^ ^1^ ^]^ self‐healing,^[^ ^2^ ^]^ and photosensitive,^[^ ^3^ ^]^ and display color variation.^[^ ^4^, ^5^, ^6^ ^]^ Conventional batteries are highly restricted by their opaqueness which prevents the realization of batteries with optical transparency that could unlock new applications including variable optical attenuator, optical switches, displays, and smart windows for energy‐efficient buildings.

The operating mechanisms of electrochromism (EC) are always accompanied by Faradaic processes, making the option of utilizing EC materials as electrodes in energy‐storage systems a growing interest over the recent decade.^[^ ^7^ ^]^ The most investigated EC materials are the transition metal oxides (TMOs) and conjugated polymers. Both classes of materials have their strengths and weaknesses. TMOs, such as tungsten oxide (WO_3_) and nickel oxide (NiO), though very stable, they exhibit moderate switching rates and are intrinsically brittle and insoluble, which limits their processibility and applications.^[^ ^5^ ^]^ On the other hand, conventional conjugated polymers (i.e., polythiophenes, polypyrrole, and polyaniline), which are made up of only organic skeletons are vulnerable to undesirable side reactions such as irreversible oxidation and nucleophilic attack by water, leading to degradation. As such, conjugated polymers are often used in tandem with organic electrolytes in encapsulated devices to preserve materials stability by preventing solvent leakage/evaporation and air‐/water‐induced oxidation reactions.^[^ ^8^, ^9^, ^10^, ^11^, ^12^, ^13^, ^14^ ^]^

While most of the organic EC materials are designed such that they can be operated with organic‐based electrolytes, those that are functionable within an aqueous medium have seen little progress. Non‐flammable, environmentally benign, and non‐toxic water‐based systems are highly desirable not only due to their safer applications but also due to convenient disposal of consumer products. Although there have been ongoing efforts toward new materials that are aqueous compatible, redox‐active metal‐coordination materials that are functional in water‐based electrolyte have rarely been explored as most of the investigations were focused on small organic molecules or organic polymers.^[^ ^14^, ^15^, ^16^, ^17^ ^]^ Unlike hydrogen bonding interactions found in many organic materials, which significantly weaken in polar aprotic solvents (such as water), coordination bondings are known to exist stably in the biological aqueous system (i.e., metalloproteins and metalloenzymes), therefore, they can likely be tailored into aqueous compatible materials.^[^ ^18^, ^19^, ^20^ ^]^

To the best of our knowledge, this class of materials has not yet been evaluated for their use in aqueous compatible electrochromic batteries. Rechargeable aqueous‐based batteries have emerged as a promising alternative to conventional batteries as the use of aqueous electrolyte not only lowers the manufacturing cost and minimizes risk for safety concerns but it also gives better ionic conductivity.^[^ ^21^, ^22^ ^]^ Amongst the aqueous‐based batteries, the use of zinc (Zn) offers several distinctive merits such as having low redox potential (−0.76 V vs SHE), excellent water stability, and being earth abundant, environmentally benign, and non‐toxic. As a multivalent charge carrier, Zn^2+^ can also provide higher energy‐density storage than commonly utilized monovalent ions (eg. Li^+^ and Na^+^).^[^ ^23^, ^24^ ^]^ In addition, Zn^2+^ containing electrolyte, unlike other transition metal ions, is colorless and transparent, which does not impede color visualization for EC applications.

In this study, a rationally designed nanostructured iron terpyridyl coordination polymer (CP) has been prepared by controlled electropolymerization with the aim of realizing electrically modulated light transmission with stable electrochemical cathodic reactions. Terpyridyl ligands are known for their strong chelation to iron compared to other first‐row transition metals (see Table S1, Supporting Information), hence they are selected in the electrochemical systems. In addition, iron exhibits a well‐defined one‐electron redox process that allows it to switch between +2 and +3 oxidation states without a change in the coordination geometry, rendering a robust system for prolonged electrochemical processes. Based on the classical Johnson–Mehl–Avrami–Kolmogorov (JMAK) interpretation, the CP has undergone a 1D growth process which can be attributed to the judicious design of the monomeric transition metal complexes. By utilizing the as‐prepared CP‐modified electrode as the cathode and a Zn anode, an integrated hybrid electrochromic Zn battery device is demonstrated, achieving high color contrast of ≈69%, fast response time of <1s, and with good endurance for over 1000 consecutive charging–discharging cycles.

## 2 Results and Discussion

### 2.1 Characterization of Electropolymerized CP on Fluorine‐Doped Tin Oxide (FTO)

**Figure 1:**
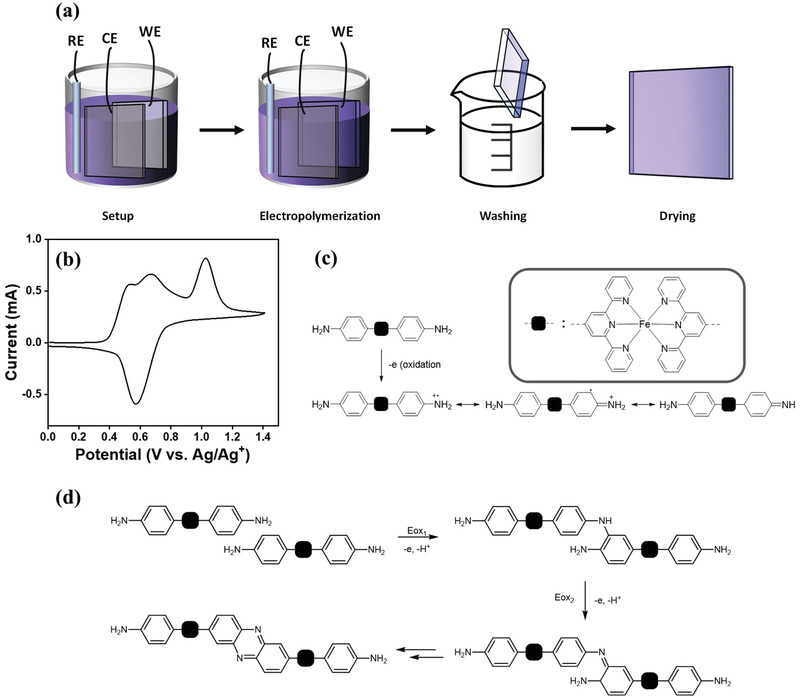
Fabrication of CP‐modified electrode. a) Schematic illustration of fabrication procedures of the CP‐modified FTO. Working electrode, counter electrode, and reference electrode are denoted as WE, CE, and RE, respectively. b) Cyclic voltammogram of the first potentiodynamic scan recorded at 100 mV s^–1^ during electropolymerization of monomeric [Fe(*p*‐tpyNH_2_)_2_]^2+^ on FTO with Pt and Ag/Ag^+^ as counter and reference electrodes. The two irreversible anodic peaks at 0.53 and 1.02 V versus Ag/Ag^+^ correspond to oxidations of the two end‐functionalized anilines. c) Resonance contributors of the radical cation formed by oxidation of aniline end group. d) Proposed intermediates and ladder‐type CP formed by electropolymerization of monomeric [Fe(*p*‐tpyNH_2_)_2_]^2+^.

In this study, an aniline end‐functionalized iron(II) terpyridyl monomer, [Fe(*p*‐tpyNH_2_)_2_](PF_6_)_2_ was synthesized and used to prepare the CP via electropolymerization (see **Figure** **1**a). During electropolymerization, the first cyclic voltammogram reveals two irreversible anodic peaks located at 0.53 and 1.02 V versus Ag/Ag^+^ (see Figure 1b), which can be attributed to the oxidation of two end‐functionalized anilines of [Fe(*p*‐tpyNH_2_)_2_](PF_6_)_2_. It is commonly believed that this process is initiated by radical cation generated by single‐electron oxidation of the diamines, which can be readily stabilized by aromatic substitution at ortho‐ and para‐position via resonance (see Figure 1c).^[^ ^25^ ^]^ In this regard, the ligand used in this work was designed in a para‐position to not only favor radical formation but also to prevent steric hindrance during polymer growth.

After electropolymerization, various characterizations have been performed to reveal the structure of the as‐prepared polymer. By comparing the FT‐IR ATR spectra of the monomer and the electropolymerized sample (Figure S2, Supporting Information), it appears that the NH_2_— stretching (3300–3500 cm^–1^) bands of the sample have disappeared after electropolymerization, indicative that both the amine groups in the monomers have reacted. A new peak at 1625 cm^–1^ has also emerged which suggests that the amine has been converted to a C═N functional group in addition to the existing pyridyl C═N stretching at 1600 cm^–1^ from the terpyridyl moiety. From the UV–vis spectra (Figure S3, Supporting Information), it shows that the metal‐to‐ligand charge transfer (MLCT) peak was shifted from 568 to 577 nm after electropolymerization, suggesting that conjugation of the coordinating ligand system has been extended, leading to lowered ligand π* orbital. Based on the information extracted from the FT‐IR and UV–vis, we herein proposed that a ladder‐like CP has formed with phenazine skeleton units as illustrated in Figure 1d. This can be further supported by the new absorption peak that appeared at ≈378 nm, which can be tentatively assigned to the *π*–*π* ^∗^ absorption contribution of the phenazine skeleton units according to the literature.^[^ ^25^, ^26^ ^]^ In addition, the ^1^H NMR spectrum and elemental analyses of the CP are also in agreement with the structural deduction from FT‐IR and UV–vis (See Figure S1c and Table S2, Supporting Information).

### 2.2 Surface Coverage of CP‐Modified Electrode

**Figure 2:**
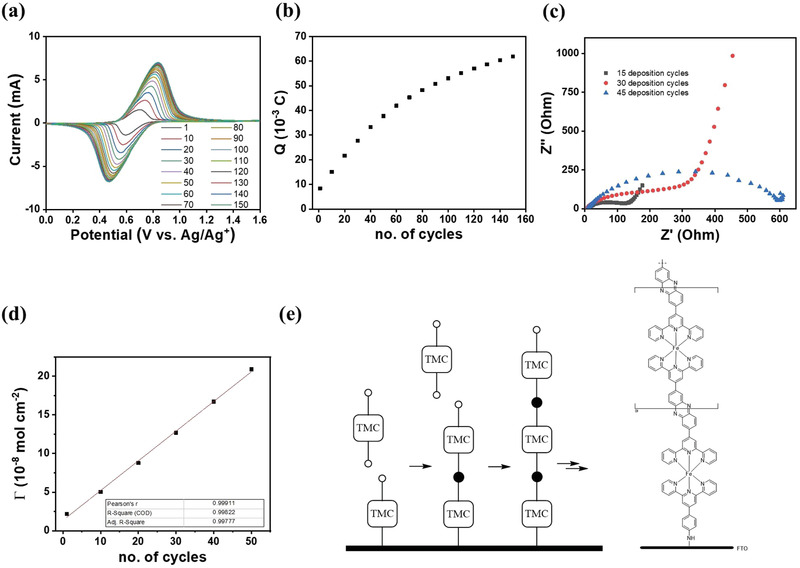
Analysis of the potentiodynamic deposition process. a) Cyclic voltammograms of 150 potentiodynamic cycles during electropolymerization. b) Changes in charge integrated over the cyclic voltammograms as a function of deposition cycles. c) EIS Nyquist plots of CP‐modified electrode fabricated by 15, 30, and 45 deposition cycles. d) Plot of *Γ* with respect to number of deposition cycles. e) Schematic illustration of 1D growth due to site‐specific reaction.

One of the advantages of electrochemical process is that the thickness of deposited CP film can be readily controlled by the number of potentiodynamic cycles and sweep rate in the cyclic voltammetric experiment. Identical cyclic voltammograms are obtained when the sweep rate and the area of the electrode are being fixed, indicative of high reproducibility of the electropolymerization process. As the CP film grows, current responses from the Fe^2+/3+^ redox couples increase concurrently, hence, it offers useful insights into the deposition process. Over 150 deposition cycles, it was found that the charge (integrated over the CV curve) would steadily increase at the initial stage and deviate from linearity at higher cycles (see **Figure** **2**a,b). The steady increase in charge and small peak‐to‐peak separation (Δ*E*_p_) between anodic and cathodic couples at the initial stage indicates that polymers exhibit a predominant capacitive response when the film is relatively thin.^[^ ^27^ ^]^

However, as CP film grows thicker, the increase in charge diminishes and the Δ*E*_p_ becomes larger. This deviation is attributable to the reducing charge transfer ability in the thicker film which can be supported by the electrochemical impedance spectroscopy (EIS) analysis that shows charge transfer ability dwindles with the number of deposition cycles (Figure 2c). The result suggests that in thicker films, some of the surface Fe^2+^ were probably not oxidized in one potential scan, hence, it hampers propagation of film growth as the polymerization is mediated by these oxidized metal centers. With that, CP‐modified electrodes were fabricated by limiting the potentiodynamic scans up to 50 potentiodynamic cycles (which is within the linearity region) to preserve the film conductivity for charge transportation.

To quantify the amount of CP that was deposited onto the electrode, the surface coverage (*Γ*, mol cm^–2^) is given as *Γ* = *Q*/*z*F*A*, where *Q* is the charge integrated over the anodic or cathodic peak in CV curve (in C), *z* is the number of electrons involved in the redox process, F is the Faraday's constant (in C mol^−1^), and *A* is the area of electrode (in cm^2^).^[^ ^28^, ^29^ ^]^ Figure 2d illustrates the plot of *Γ* of CP‐modified electrode against the number of cycles up to the first 50 deposition cycles.

### 2.3 Growth Analysis Based on Johnson–Mehl–Avrami–Kolmogorov (JMAK) Theory

In order to decipher the CP growth mechanism, classical Johnson–Mehl–Avrami–Kolmogorov (JMAK) theory was used to provide insight into the phase transformation of the electropolymerization process.^[^ ^30^, ^31^, ^32^ ^]^ In the analysis, time‐dependence areal capacity from the cyclic voltammogram was used to evaluate the Arvrami parameters, specifically, the Avrami exponent (*n*) which gives information on growth dimensionality. From the double logarithm plot (see Figure S4b, Supporting Information), *n*‐value of 1.19 (or ≈1) was derived from the slope, suggesting that the film growth has taken place in an anisotropic 1D fashion. This is in line with the end‐to‐end reaction of the electropolymerization process (see Figure 2e), to which the reaction occurs only at specific sites (or functional groups) rather than being a randomized process.

### 2.4 Electrochemical Characterization of CP‐Modified Electrode

Unlike most of the CPs where redox properties originate from the ligand, the as‐prepared Fe‐based CP exhibits a robust metal‐centered redox process, therefore, it is envisioned to possess an exceptional electrochemical stability.^[^ ^33^, ^34^, ^35^ ^]^ To simultaneously explore the uses of this CP‐modified electrode for both EC and Zn‐based energy storage applications, spectroelectrochemical and electrochemical properties of the as‐prepared electrode were characterized with Zn serving as both counter and pseudo‐reference electrode. In this study, high concentration 3 m aqueous Zn(ClO_4_)_2_ was used as the supporting electrolyte to suppress water electrolysis and stretch the potential window up to 2.3 V versus Zn/Zn^2+^.

**Figure 3:**
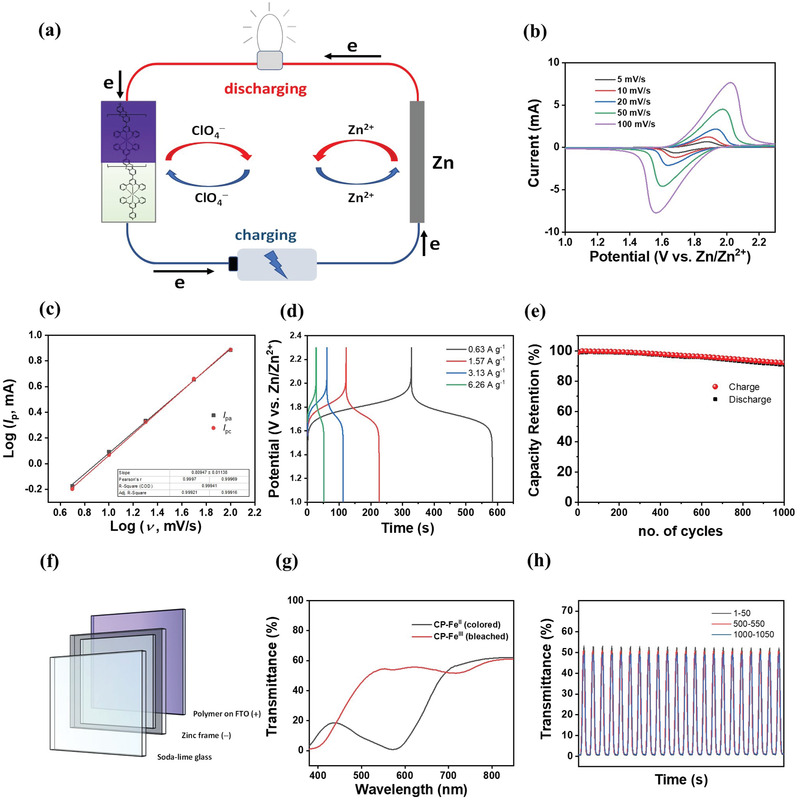
Electrochemical and spectroelectrochemical performances of CP‐modified FTO. a) Schematic representation of a hybrid electrochromic zinc battery. b) Cyclic voltammograms of CP‐modified electrode at different scan rates with Zn as both counter and pseudo‐reference electrodes in 3 m Zn(ClO_4_)_2_. c) Plot of log *i* against log *v* to derive *b*‐value for analysis. d) Galvanostatic charge–discharge curves for CP‐modified electrode in the potential range of 1–2.3 V versus Zn/Zn^2+^ under different current density (ranging from 0.63 to 6.26 A g^–1^). e) Capacity retention plot over 1000 cycles at current density of 6.26 A g^–1^. f) Schematic illustration of the device testing configuration. g) Spectral transmittance of the device recorded at 1 V (black) and 2.3 V (red). h) Changes in transmittance of the device at 572 nm of recorded over 1000 consecutive cycles.

Over the cyclic potential range between 1 and 2.3 V (vs Zn/Zn^2+^), the CP‐modified FTO electrode (prepared by 20 potentiodynamic deposition cycles) exhibited one pair of pronounced redox couple, indicative of typical Faradaic behavior.^[^ ^36^ ^]^ The cathodic and anodic peaks of the redox couple correspond to oxidation of Fe^2+^ metal and reduction of Fe^3+^ metal centers, respectively. With an increased sweep rate from 5 to 100 mV s^–1^, the current response of these redox couples gradually increased, alongside larger *ΔE*_p_ as illustrated in **Figure** **3**b. It is commonly known that scan‐rate dependency current response either originates from surface capacitive effect or diffusion‐controlled processes (e.g., anion movement for electroneutrality).^[^ ^37^ ^]^

To evaluate the origin of the current response, plot of *i*_p_ against *v* and *v* ^1/2^ was charted as illustrated in Figure S6, Supporting Information. Both of the plots show good linear relationships, suggesting that the current response is a combination of capacitive effect and diffusion‐controlled process. Further evaluation of individual contributions was made using the Dunn method^[^ ^37^ ^]^ (*i* = *av^b^*), to which the *b*‐value of both anodic and cathodic current show a value of ≈0.8 (see Figure 3c), indicating that the current response is predominantly a capacitive effect (0.8−1 is considered capacitive according to Dunn's definition).^[^ ^37^ ^]^ A plot of peaks potential *E*_ox_ (and *E*_red_) against log *v* (see Figure S7, Supporting Information) shows that beyond the critical scan rate of 8.9 mV s^–1^ for anodic process (or 7.0 mV s^–1^ for cathodic process), the contribution from the diffusion‐controlled process will start taking effect.^[^ ^38^ ^]^ Nevertheless, sharp redox peaks are preserved even at a high scan rate of 100 mV s^–1^ due to the predominant capacitive current response, indicative of rapid charge transfer process which is critical for EC response rate. Moreover, the highly symmetric redox peaks at different scan rates reflect excellent electrochemical stability of this CP‐modified electrode.

To illustrate the energy‐storage feasibility of this CP‐modified electrode, it was quantitatively analyzed by galvanostatic charge–discharge (GCD) experiments (see Figure 3d). From the GCD, the gravimetric, volumetric, and areal capacity of the CP‐modified electrode can achieve up to 57.3 mAh g^–1^, 263.6 mAh cm^–3^, and 14.5 mAh m^–2^ at current densities of 0.63 A g^–1^, 3.64 mA cm^–3^, and 0.02 mA cm^–2^ respectively. Moreover, the CP‐modified electrode also exhibits excellent rate capability, to which high gravimetric (54.1 A g^–1^), volumetric (249.1 mA cm^–3^), and areal capacities (13.7 mAh m^–2^) could be retained even when the current density was increased tenfold to 6.26 A g^–1^, 36.36 mA cm^–3^, and 0.20 mA cm^–2^ respectively (see Figure S8, Supporting Information). This is in line with the capacitive behavior of the electrode which shows good charge–transfer kinetics. Table S3, Supporting Information, summarizes the performance of current state‐of‐the‐art EC energy storage materials. It shows that the CP‐modified electrode exhibits comparable capacity to many inorganic materials while having better EC performance. In addition, energy storage performances are well preserved even in a prolonged electrochemical cycling test, in which 91% capacity retention with virtually 100% coulombic efficiency was achieved after 1000 consecutive cycles as illustrated in Figure 3e.

### 2.5 Spectroelectrochemical Characterization of CP‐Modified Electrode

In order to assess the EC performance of the as‐prepared electrodes, in situ spectroelectrochemical spectra were recorded spanning the entire visible‐light spectrum from 380 to 850 nm. The analysis reveals that the CP's transmittance at 572 nm was dynamically increased when the potential was stepped up from 1.0 to 2.3 V (vs Zn/Zn^2+^), and it can revert to its original value when the potential was cycled back to 1.0 V (vs Zn/Zn^2+^, Figure 3g). This causes the material to change its absorption behavior between the initial purplish‐blue state and the bleached transparent state. The electrochromic changes can be attributed to the suppression of the MLCT transition band after one electron is removed from the HOMO energy level of the metal center during an anodic scan. The energy gap between the “HOMO−1” and LUMO is much larger, causing it to take place at a shorter wavelength outside the visible region or simply it would not occur due to the large bandgap.

The EC performances, particularly, the optical contrast (Δ*T* = *T*_bleaching_ − *T*_coloration_) at 572 nm was being studied under different thicknesses controlled by the number of deposition cycles (Figure S9 and Table S4, Supporting Information). It was found that the Δ*T* will first drastically increase and then gradually decrease. While the increase in Δ*T* can easily correlate to the increasing amount of EC materials on the electrode, the eventual drop in Δ*T* is not intuitive and requires further investigation. The surface morphology of the CP‐modified electrodes was evaluated using scanning electron microscopy (SEM) and atomic force microscopy (AFM). It was found that as the CP‐film grows thicker, the polymers tend to aggregate with their neighboring polymers and form clusters (Figure S10, Supporting Information). The tendency of aggregation could be arising from the ladder‐type linkages within the CP, resulting in a slanted growth. Although the aggregates are not visually perceived, they would interfere with the light passing through, causing a decrease in *T*_bleaching_ (Table S4, Supporting Information). Another reason for lower Δ*T* could be a result of poorer charge‐transfer ability in thicker film, leading to partial oxidation of the EC materials. AFM analysis shows that the root mean square (RMS) roughness of the surface rose from only 1.916 to 27.209 nm from 10 to 50 deposition cycles (Figure S10, Supporting Information), indicative that uniformity will reduce with the number of deposition cycles.

From the result of the EC switching, Δ*T* of as high as 69% can be attained at 572 nm with sub‐second switching rate (*t*_bleaching_ = 0.85 s and *t*_coloration_ = 0.5 s) and coloration efficiency of up to 321 cm^2^ C^–1^ (see Figure S11, Supporting Information). This is better than most thin‐film EC materials used for energy storage application as shown in Table S3, Supporting Information. The electrochromic endurance of the CP modified electrodes was further assessed via changes in transmittance over the number of EC switching cycles between bleached and colored states. It was found that the film was able to retain ≈91% of its initial Δ*T* after consecutive 1000 electrochemical cycles (see Figure S12, Supporting Information), which is in line with its capacity retention. This illustrates that the CP‐modified electrode has excellent endurance as an EC energy storage electrode.

In correlation with the electrochemical and optical information, insights into the frontier molecular orbitals were obtained and it was found that the energy of HOMO and LUMO is −5.0 and −3.5 eV, respectively. The derived bandgap of 1.5 eV suggests that the polymer behaves like a conjugated polymer even if it possesses an interrupted conjugation system due to metal inclusion. This is consistent with the capacitive behavior of the electrode which shows good charge–transfer kinetics.

## 3 Fabrication of Device

**Figure 4:**
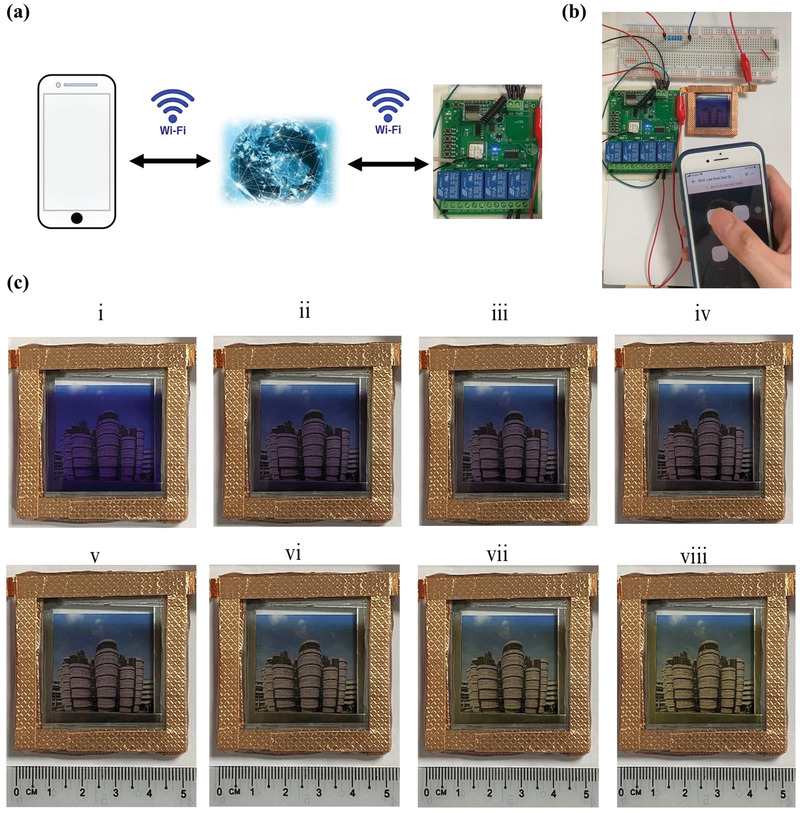
Remote control of the electrochromic device. a) The route map between the user and the remote‐controlled switch. b) Actual setup of WiFi remote‐controlling EC system. c) Photograph of 50 × 50 mm device captured during the color changing process from i) colored to viii) bleached state.

Zn is opaque and therefore it blocks the light from transmitting through. In order to allow light to pass through for color visualization while preserving zinc as the anode in the system, Zn has been shaped into a frame and configured into a device. Unlike many EC devices that show significant drops in performance in the device setting,^[^ ^39^ ^]^ our device shows essentially similar spectrochemical performance to that of cuvette measurement as we have directly performed all the analyses in a two‐electrode setup (Figure 3f–h; Figure S12a–c, Supporting Information). To demonstrate the capability to remotely control the EC process, the device was connected to an integrated WiFi circuit board which acts as a switch, that can be wirelessly turned “ON” and “OFF” by using a mobile phone app as illustrated in **Figure** **4**; Movie S1, Supporting Information.

## 4 Conclusion

We have demonstrated an asymmetric electrochromic zinc battery with anodic CP‐modified electrode prepared via highly reproducible electropolymerization. With the advances in EC materials coming to maturity, the dominating issue that needs to be addressed falls on the efficient film production in industrial setting. We show that the as‐prepared electrodes can be facilely manipulated by electrochemical parameters, resulting in modified electrodes with different surface morphology and thickness. These can effectively affect optical and charge‐transport performances of the electrodes, which heavily influence their optical and electrochemical performances. The current work also shows that CP is a promising candidate for multifunctional EC materials operatable in an aqueous medium, allowing a safer application for consumer usage. With the effort and progress in transparent, flexible, and stretchable polymeric substrate, such as recent progress in bendable EC devices using ITO‐coated PET films,^[^ ^40^, ^41^ ^]^ it is envisioned that by soft electrochromic energy storage device can be realized when these deformable substrates are used.^[^ ^42^, ^43^, ^44^ ^]^

## Conflict of Interest

The authors declare no conflict of interest.

## Supporting information

Supporting Information

Supplemental Movie 1

## Data Availability

The data that supports the findings of this study are available in the Supporting Information of this article.
