# Supplementary material for: Electropolymerized 1D Growth Coordination Polymer for Hybrid Electrochromic Aqueous Zinc Battery
Source: Adv Sci (Weinh). 2021 Sep 17;8(21):2101944. doi: 10.1002/advs.202101944 (PMC8564436; doi:10.1002/advs.202101944)
Supplement: Supplementary file 1 — Supporting Information [file ADVS-8-2101944-s001.pdf]

WILEY-VCH

© 2021 Wiley-VCH GmbH

ADVANCED SCIENCE
Open Access

Supporting Information

for *Adv. Sci.*, DOI: 10.1002/advs.202101944

Electropolymerized One-dimensional Growth Coordination Polymer for Hybrid Electrochromic Aqueous Zinc Battery

*Wei Church Poh, Xuefei Gong, Fei Yu, Pooi See Lee**

Supporting Information

Electropolymerized One-dimensional Growth Coordination Polymer for Hybrid Electrochromic Aqueous Zinc Battery

*Wei Church Poh, Xuefei Gong, Fei Yu, Pooi See Lee**

Reagent and Materials

4-aminophenylboronic acid pinacol ester (97 %), tetrakis(triphenylphosphine)palladium(0) ($Pd(PPh_3)_4$, 99%), sodium carbonate (Na_2CO_3, 99.5%) and iron(II) acetate ($Fe(OAc)_2$, 95%) ammonium hexafluorophosphate (NH_4PF_6, >95%) were purchased from Sigma-Aldrich. 4'-Bromo-2,2':6',2''-terpyridine (95-98%) was obtained from Fluorochem and was recrystallized before use. Solvents used in synthesis including dimethoxyethane (DME), ethanol (EtOH), acetic acid (AcOH), and dichloromethane (CH_2Cl_2) were of analytical or HPLC grade and were used as received. Fluorine doped tin oxides (FTO) were cleaned by sequential sonication in H2O, acetone and isopropyl alcohol, and blew dry using a compressed airgun.

NMR spectroscopy

1H NMR spectra were obtained from Bruker Avance DPX-400 (400 MHz) Fourier-transform NMR spectrometer at 298 K and calibrated against tetramethylsilane, $Si(CH_3)_4$.

Fourier-Transform Infrared (FT-IR) Spectroscopy

All FTIR spectra were collected using PerkinElmer Fourier Transform Infrared (FTIR) Frontier spectrometer with attenuated total reflection (ATR) which average over 32 scans at a resolution of 4 cm^{-1}.

Electronic Absorption and Emission Spectroscopy

The electronic absorption spectra in this work were acquired using a Perkin Elmer Lambda 950 spectrophotometer in transmittance mode. In-situ spectroelectrochemical measurements were performed by applying potential using an Autolab potentiostat. The emission profile of the complex was recorded using Cary Eclipse Spectrophotometer excite at 572 nm.

Electrochemical Measurements

All electrochemical experiments and characterization such as cyclic voltammetry (CV), electrochemical impedance spectroscopy (EIS), and galvanostatic charge-discharge (GCD) were performed using an Autolab PGSTAT30. EIS information was obtained by applying an AC voltage of 5mV amplitude spanning a frequency range between 0.1 and 100k Hz.

Surface Morphology

Scanning electron microscopy (SEM) and atomic force microscopy (AFM) images were acquired from a JOEL 7600F SEM and Asylum Research Cypher S AFM.

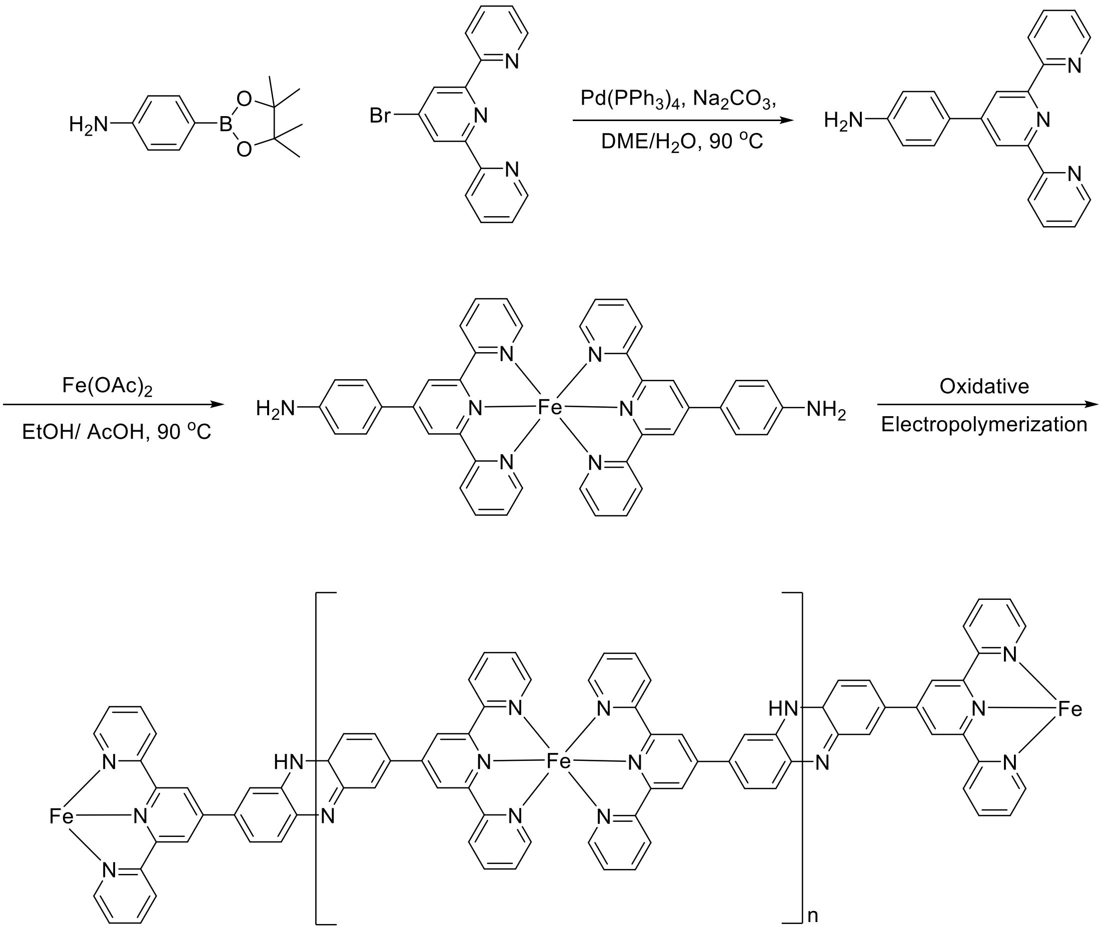

Scheme S1 Synthetic pathway for the CP.

4-([2,2':6',2''-terpyridin]-4'-yl)aniline, *p*-tpyPhNH$_2$

This was synthesized based on the classical Suzuki-Miyaura coupling reaction in a heterogeneous mixture of DME and H_2O in a Schlenk flask. Pd(PPh$_3$)$_4$ (278 mg, 0.24 mmol) was added to a suspension of 4'-bromo-2,2':6',2"-terpyridine (1000 mg, 3.2 mmol), 4-aminophenylboronic acid pinacol ester (842 mg, 3.8 mmol) and Na_2CO_3 (1019 mg, 9.6 mmol) in degassed 60 mL DME-H_2O (5:1 v/v). The resulting mixture was heated to 90 ºC for 24 hours in the sealed flask. After which, the mixture was washed with saturated aqueous NH_4Cl, brine and H_2O and extracted with CH_2Cl_2. After removal of solvent, the crude product was recrystallized in boiling EtOH to give product as pale pink solid. Yield: 820 mg, 2.5mmol, 79%. ^{1}H NMR (400 MHz, $CDCl_3$, 298K, δ/ ppm): δ 8.73 (d, J = 4.8 Hz, 2H), 8.69 (s, 2H), 8.66 (d, J = 7.9 Hz, 2H), 7.87 (td, J = 7.7, 1.8 Hz, 2H), 7.78 (d, J = 8.6 Hz, 2H), 7.34 (td, J = 6.2, 1.2 Hz, 2H), 6.80 (d, J = 8.6 Hz, 2H), 3.86 (s, 2H).

[Fe(*p*-tpyPhNH$_2$)$_2$](PF$_6$)$_2$

[Fe(*p*-tpyNH$_2$)$_2$](PF$_6$)$_2$ was prepared by mixing *p*-tpyPhNH$_2$ ligand (200mg, 0.62 mmol) and Fe(OAc)$_2$ (51mg, 0.29 mmol) in 25 ml degassed EtOH-AcOH (4:1 v/v). After heating at 90 ºC for 24 hours, the mixture was diluted with 25 ml water and 2 ml of 0.25 g/ml NH_4PF_6 was added dropwise to induce precipitation of dark purple solid. The precipitate was then filtered and washed with H_2O and Et_2O to give purple solid product. ^{1}H NMR (400 MHz, CDCN, 298K, δ/ ppm): δ 9.07 (s, 2H), 8.57 (d, J = 7.9 Hz, 2H), 8.12 (d, J = 8.5 Hz, 2H), 7.87 (t, J = 6.6 Hz, 2H), 7.18 ((d, J = 5.3 Hz, 2H), 7.05 (t, J = 6.5 Hz, 2H), 6.99 (d, J = 8.4 Hz, 2H). 13C NMR (100 MHz, CDCN, 298K, δ/ppm): δ 160.9, 159.4, 154.1, 152.2, 151.5, 139.5, 130.0, 128.1, 125.2, 124.5, 120.8, 115.9. FT-IR (ATR): 3488 and 3395 cm^{-1} v(NH$_2$ stretches).

Fabrication of CP-modified Electrode and Hybrid EC Battery Device

CP was grown on top of pre-cleaned FTO by using a three-electrode setup with FTO as working electrode (WE), Pt as counter electrode (CE), and Ag/Ag$^+$ (0.01 M $AgNO_3$ in 0.1 M tetrabutylammonium perchlorate (TBAP)) as the reference electrode (RE) using potentiodynamic scans at 100 mV s^{-1}. The electrolyte used comprised of 1 mM of [Fe(tpyPhNH$_2$)$_2$](PF$_6$)$_2$ mixed with 0.1 M TBAP as the supporting electrolyte in acetonitrile. After that, the as-prepared electrode was cleaned by sonicating the electrode in acetonitrile for 30 seconds.

Hybrid EC battery devices were prepared by sandwiching a Zn frame between a transparent soda-lime glass and the CP-modified electrode. VHB tape (3M) was used as a spacer between the Zn and the CP-modified electrode allowing electrolyte containment.

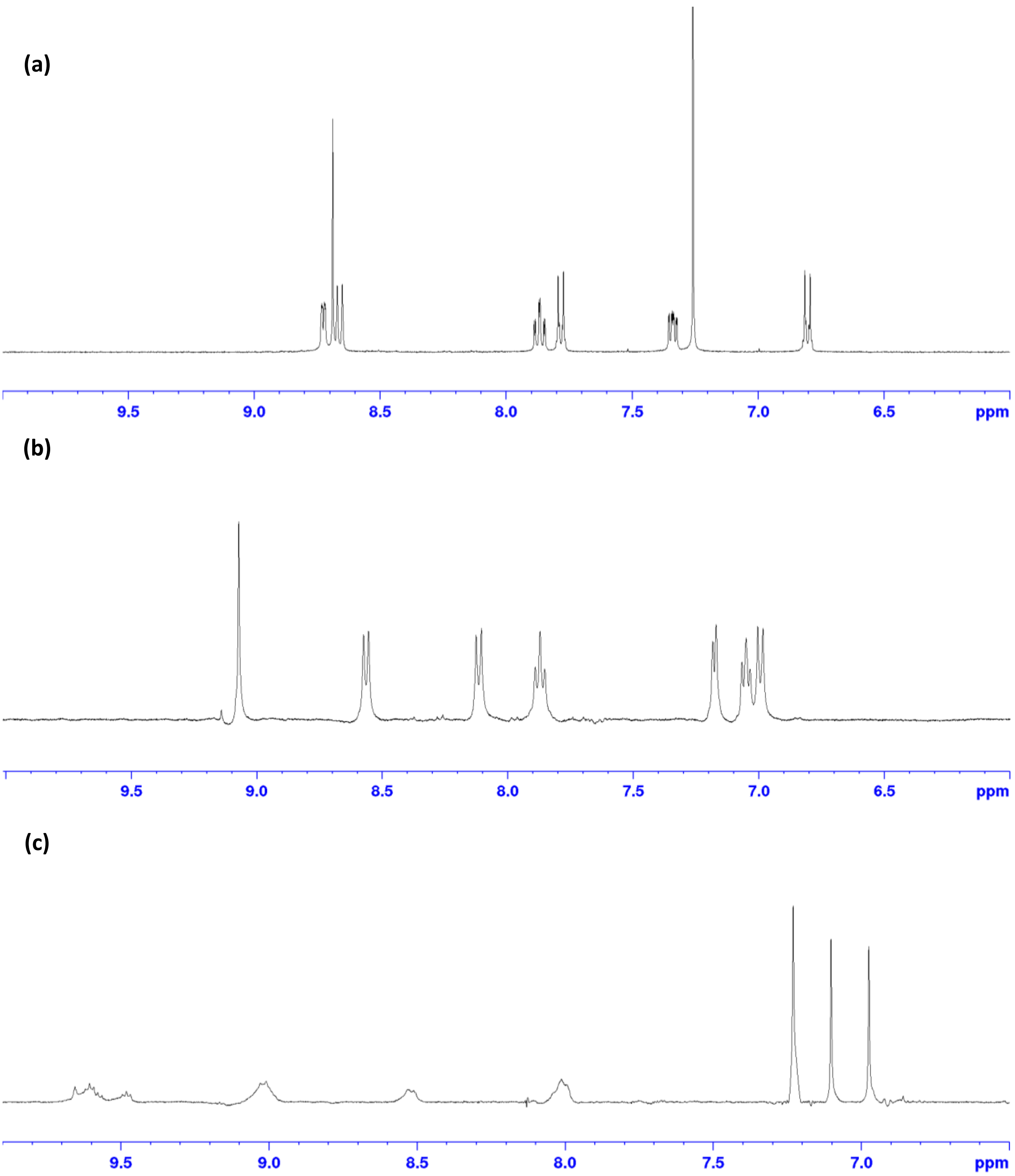

Figure S1 NMR spectra**.** ^{1}H NMR of (a) *p*-tpyPhNH$_2$, (b) [Fe(*p*-tpyPhNH$_2$)$_2$](PF$_6$)$_2$, (c) electropolymerized CP.

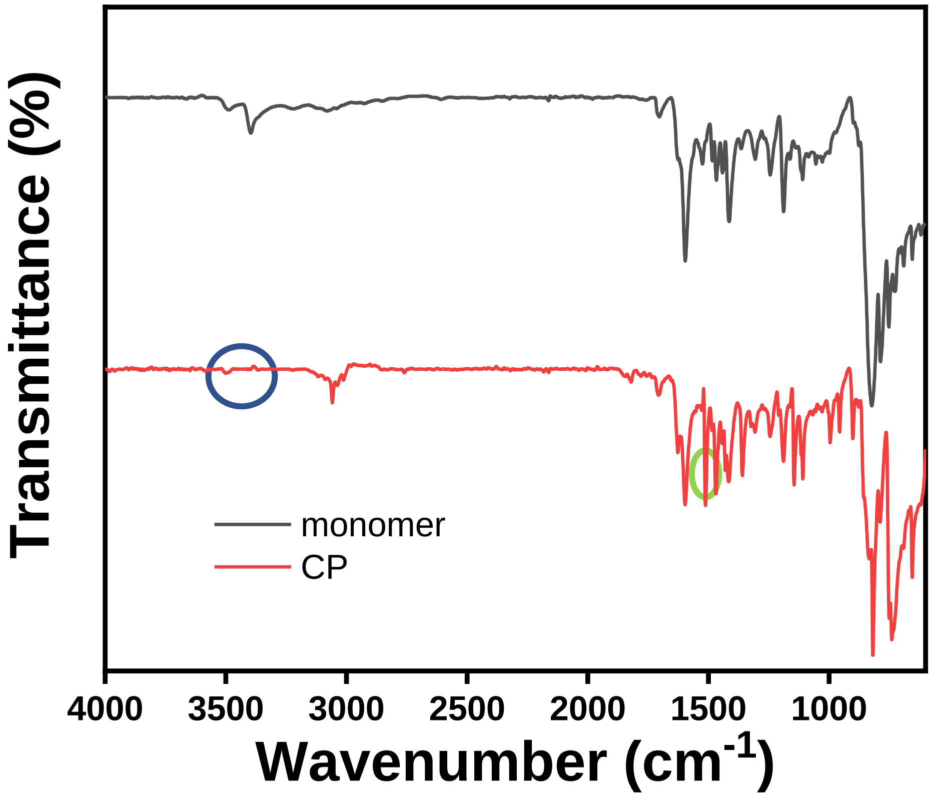

Figure S2 FT-IR spectra of [Fe(*p*-tpyPhNH$_2$)$_2$](PF$_6$)$_2$ and its electropolymerized CP. After electropolymerization, the NH$_2$ (blue) stretching peaks became vanished with the appearance of a new phenazine C=N (green) stretching peak.

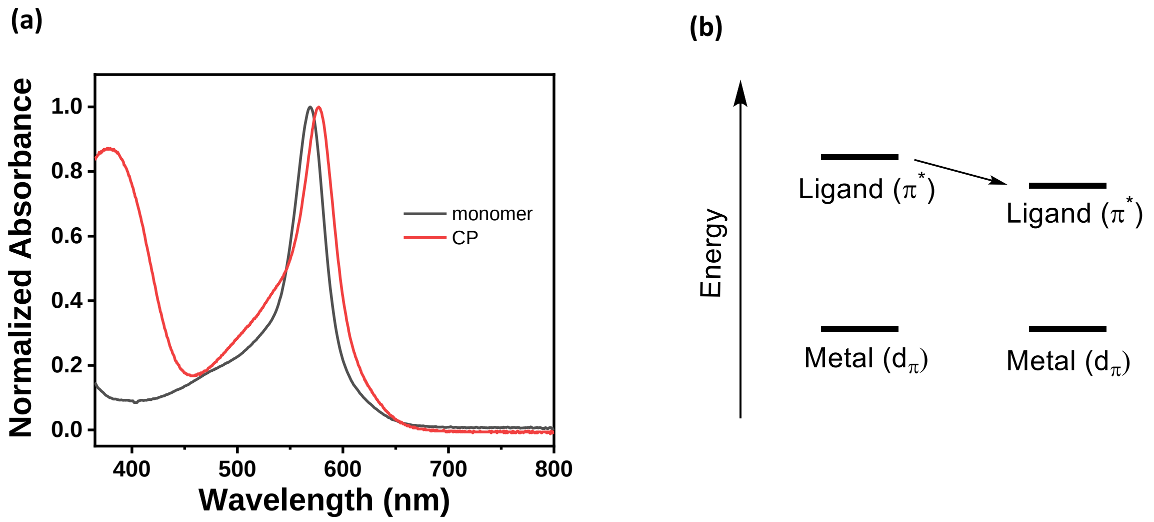

Figure S3. (a) UV-Vis Spectra of [Fe(*p*-tpyPhNH$_2$)$_2$](PF$_6$)$_2$ and its electropolymerized counterpart on FTO substrate. (b) Energy level diagram showing the effect of increased conjugation. (The effect on the metal d$_\pi$ is negligible hence, omitted here)

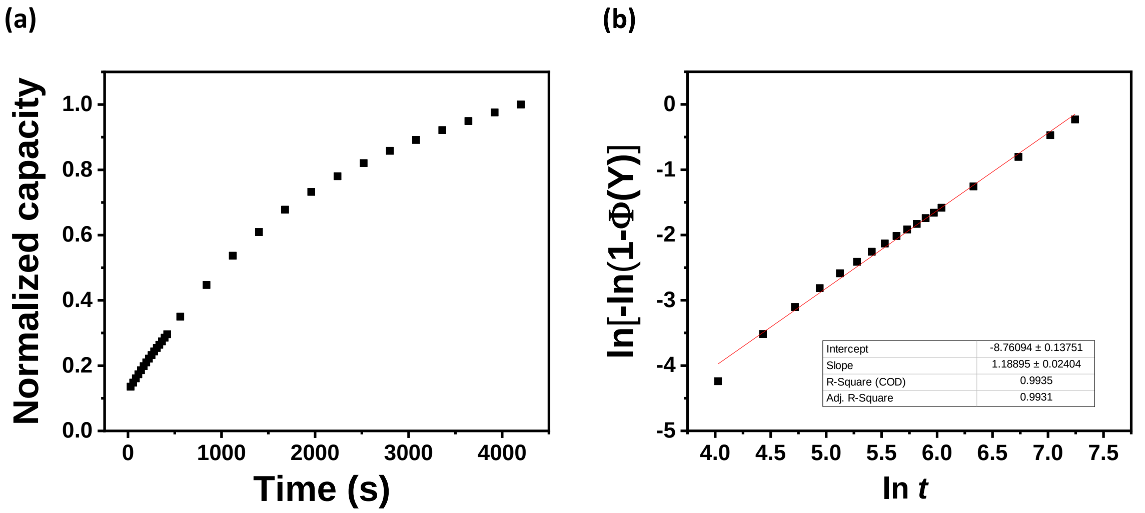

Figure S4. Time-dependence parameters for Johnson–Mehl–Avrami–Kolmogorov (JMAK) interpretation. (a) Normalized areal capacity with respect to time, showing two stages: linear and non-linear regions. (b) The initial stage where reflect the growth most accurately was presented in a double logarithmic plot of $-\ln(1 - X)$ vs. t to derive the Avrami parameter for analysis.

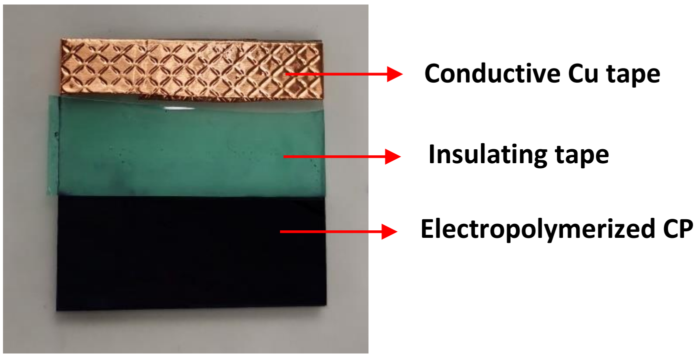

Figure S5. Photograph of an FTO with the electroactive surface was controlled by an insulating tape.

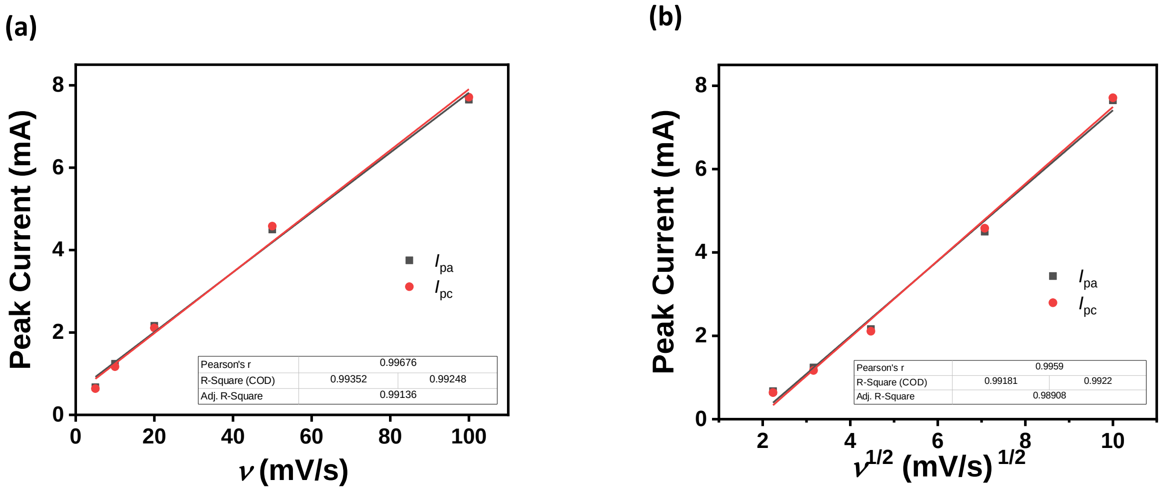

Figure S6. Electrochemical characterization of the CP-modified FTO with Zn acting as counter and pseudo-reference electrodes. Plot of i_p against (a) υ and (b) $\upsilon^{1/2}$ for analysis of charge transfer behavior.

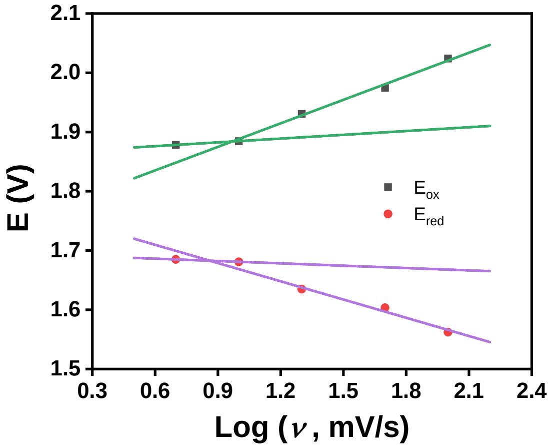

Figure S7. Plot of E_{ox} and vs log ν to evaluate critical scan rate to which semi-infinite diffusion comes into effect.

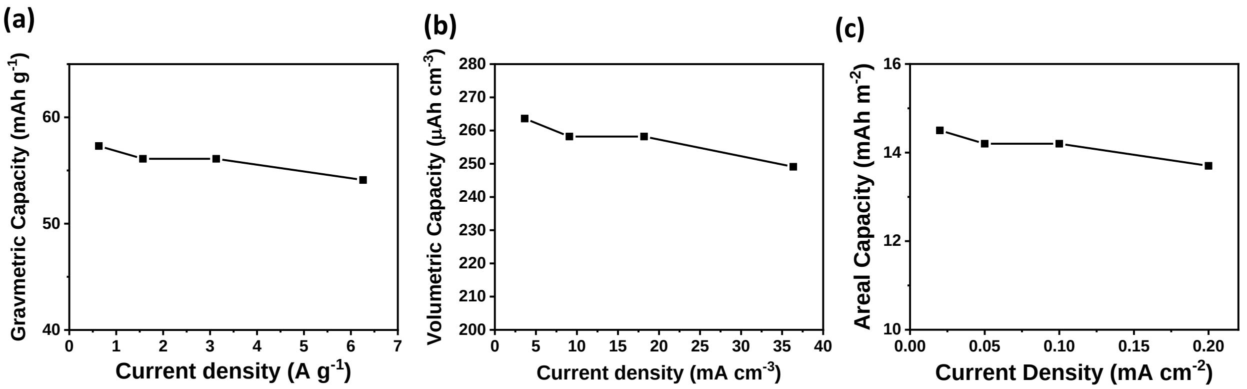

Figure S8. Evaluation of rate capability of the CP-modified FTO. (a) Gravimetric, (b) volumetric, and (c) areal capacities of CP-modified electrode as a function of current density show minimal variation, indicative of good rate capability.

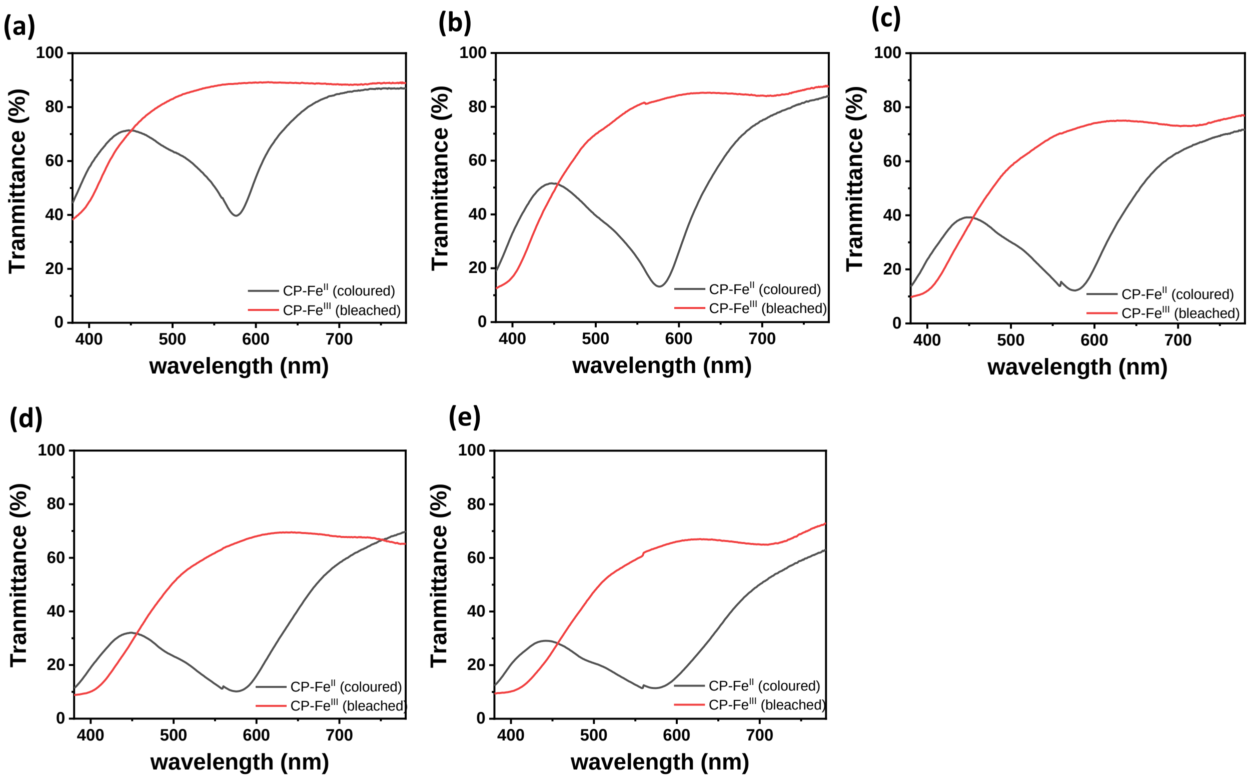

Figure S9. Spectroelectrochemical profiles of varied thickness controlled by deposition cycles (a) 10, (b) 20, (c) 30, (d) 40, and (e) 50. (*Instrumental artifacts can be seen at 560 nm, but this will not affect our interpretation)

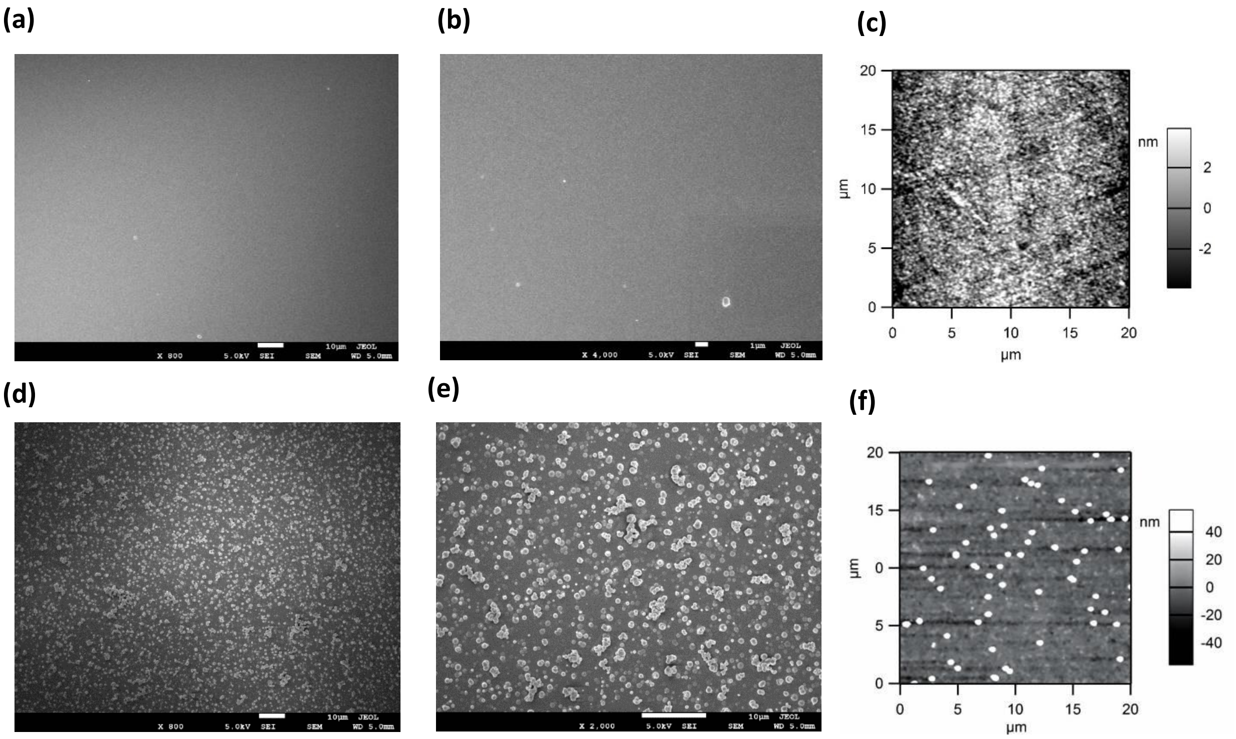

Figure S10. Surface morphology of CP-modified electrodes. SEM images of CP-modified electrodes at different magnifications fabricated by (a, b) 10 and (d, e) 50 potentiodynamic cycles. AFM surface topography of the CP modified electrodes fabricated by (c) 10 and (f) 50 potentiodynamic cycles.

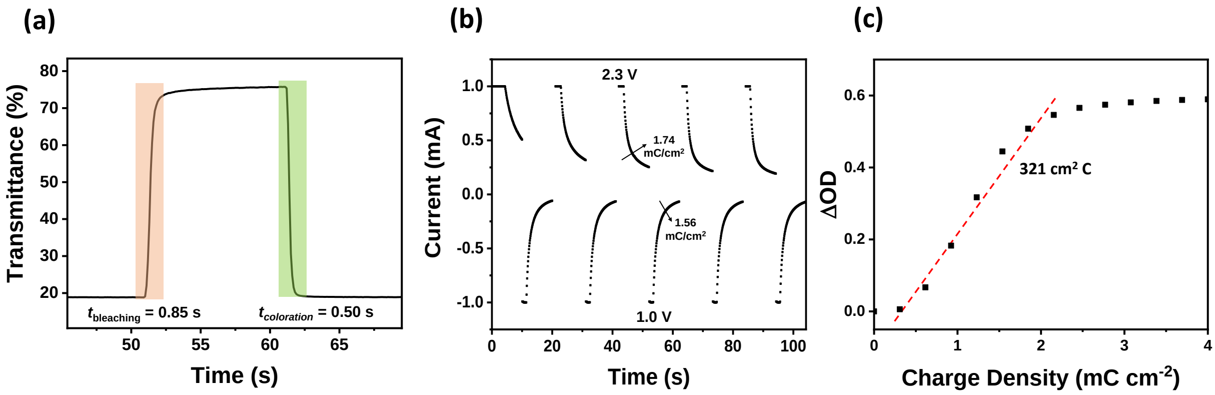

Figure S11. (a) Response times (defined here as the time required for 90 % change in maximum ΔT) for coloration ($t_{\text{coloration}}$) and bleaching ($t_{\text{bleaching}}$) derived from spectroelectrochemical measurement at 572 nm with potential applied between 1.0 V (red) and 2.3 V (green) at 10 s intervals. (b) Charge/discharge amount by the CP film between 1.0 V and 2.3 V is calculated by integrating the area under the curve of the chronoamperometric experiment. (c) Optical density variation with respect to charge density at 572 nm is used to derive the coloration efficiency, η. ($\eta = log(T_b/T_c)/Q_d$, where T_b and T_c are bleached and colored transmissive value, and Q_d is the charged consumed per unit area)

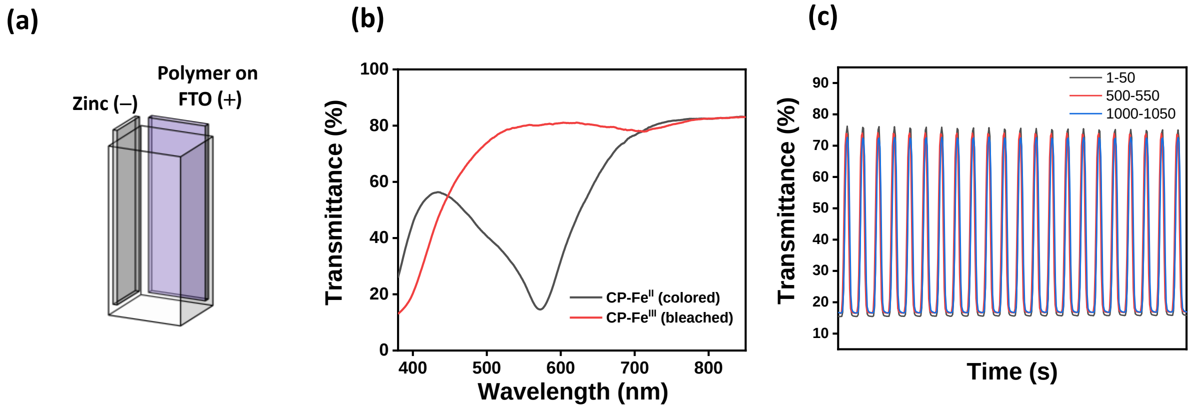

Figure S12. Spectroelectrochemical performances of CP-modified FTO. (a) Schematic illustration of a cuvette testing configuration. (b) Spectral transmittance of the cuvette recorded at 1 V (black) and 2.3 V (red). (c) Changes in transmittance of the cuvette at 572 nm of recorded over 1000 consecutive cycles.

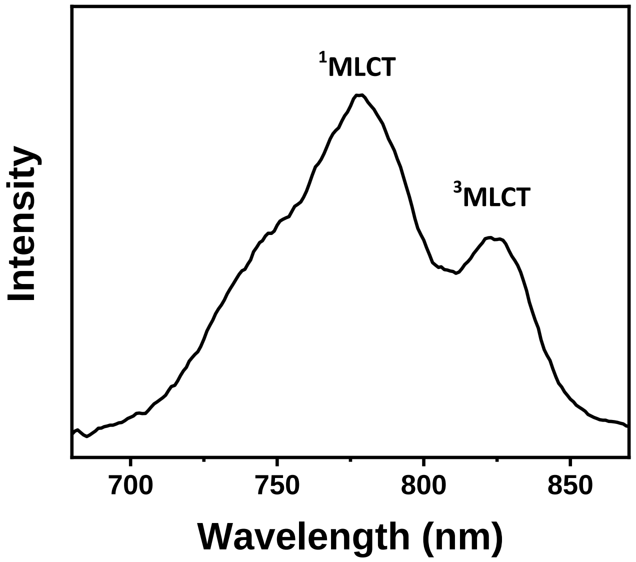

Figure S13. Emission spectrum of CP-modified electrode excited at 572 nm displays two peaks which can be tentatively assigned to ^{1}MLCT and ^{3}MLCT respectively.

Table S1. Physical properties of metal-terpyridine system.[1–3] ([a] Measurement is determined in acetonitrile.)

Element	Association constant of mono(tpy) complexation $[MtpyLn]^{2+}$ (Log Ka_1)	Association constant of bis(tpy) complexation $[Mtpy_2]^{2+}$ (Log Ka_2)	Ligand self-exchange half-life ($[[Mtpy_2]^{2+}$ with tpy] (min)	Formation rate constant of $[MtpyLn]^{2+}$ Log (M^{-1} s^{-1})	Formation rate constant of $[Mtpy_2]^{2+}$ Log (M^{-1} s^{-1})
**Fe (II)**	7.1	13.8	8400 (35.4 ºC)<br>2730 (45.8 ºC)	4.75 (25 ºC)	7.04 (5 ºC)
**Cu (II)**	12.3	6.8	<0.1 (0.1 ºC)	7.30 (6.5 ºC)	-
**Zn (II)**	6<br>8.2[a]	6.1[a]	<0.1 (0.1 ºC)	6.04 (25 ºC)	-
**Ni (II)**	10.7	11.1	610 (44.8 ºC)	3.15 (25 ºC)	4.88 (10 ºC)
**Co (II)**	9.5<br>8.4	9.1<br>9.9	19 (10.5 ºC)	4.38 (25 ºC)	6.70 (5 ºC)

Table S2. Elemental analyses of the electropolymerized CP.

	C	H	N
Found %	51.15	23.04	11.34
Calculated %	51.03	23.06	11.34

Table S3. The performance of current state-of-the-art EC energy storage materials.

**Material (WE/CE)**	**Electrolyte**	**Fabrication method**	**ΔT (%)**	**t_b/t_c (s)**	**η (cm^2 C^{-1})**	**Capacity (max)**	**EC battery endurance**	**Ref.**
WO_3 nanowires/ rGO-NiO	0.1 g/mL $LiClO_4$	Chemical bath deposition/ Electrophoretic deposition	43	2.6/2.5	135.5	75 mAh g^{-1}	86.7% (2500 cycles)	[4]
$MoO_3W_{0.71}Mo_{0.29}O_3$/ FTO	1 M $LiClO_4$	Spray-coating	49.6	>20	20.8	41.9 mAh g^{-1}	25% (100 cycles)	[5]
NiO/ Pt	1 M KOH	Spin-coating	63.6	9.5/11.5	42.8	42.7 mAh g^{-1}	56.4% (5000 cycles)	[6]
$W_{0.71}Mo_{0.29}O_3$/ Zn	1 M $ZnSO_4$	Drop-casting	76	-	-	166 mAh g^{-1}	53% (100 cycles)	[7]
WO_3/ Zn	1 M $ZnSO_4$/ $AlCl_3$	Electrodeposition	77	5.7/10.3	-	126.3 mAh/m^2	57% (200 cycles)	[8]
WO_3-PEDOT/PSS/ CeO_2/TiO_2	0.5 M H_2SO_4	Inkjet Printing	73.3	15.8/12.7	108.9	~65 mAh/m^2	45.2 % (1000 cycles)	[9]
NA/HP_2W_{18}/ Al	3 M KCl	Printing method	-	-	-	8.01 mAh g^{-1}	-	[10]
Ni-BTA/ Pt	1 M KOH	Chemical bath deposition	58.3	5/1.8	179.7	168.1 mAh g^{-1}	90 % (1000 cycles)	[11]
Symmetric WO_3-PPy or WO_3-MnO_2	0.5 M H_2SO_4/ PVA	Electrodeposition and Electropolymerization	~29	-	-	~55 mAh/m^2 (WO_3-PPy) ~125 mAh/m^2 (WO_3-MnO_2)	-	[12]
PANI/ Al	5 M $Al(TOF)_3$/ 1 M H_3PO_4	Drop-casting	59	-	84	225 mAh g^{-1} (C cloth) 59 mAh g^{-1} (on FTO)	80.8% (500 cycles) 58% (3850 cycles)	[13]
PB/ Al	3 M KCl	Electrodeposition	52.2	4.1/4.6	-	75 mAh g^{-1}	81% (50 cycles)	[14]
W18O49-PANI/ Al		Spin-coating and Electropolymerization	31	-	45.68	52.96 mAh g^{-1}	-	[15]
PPy/ Al	3 M KCl	Electrodeposition	59	6.5	-	75.25 mAh g^{-1}	92% (50 cycles)	[16]
Fe-CP/ Zn	3 M $Zn(ClO_4)_2$	Electropolymerization	69.1	0.85/0.5	321	57.3 mAh g^{-1}	91% (1000 cycles)	**This work**

Table S4. Optical Information recorded at 572 nm of the CP-modified electrode at different deposition cycles.

Deposition cycles	$T_{coloration}$	$T_{bleaching}$	ΔT (%)
10	40.4	88.6	48.2
20	13.2	82.4	69.2
30	12.2	71.5	59.3
40	10.3	65.1	54.8
50	11.4	63.4	52.0

Supplementary References

[1] R. W. Lewis, N. Malic, K. Saito, R. A. Evans, N. R. Cameron, *Chem. Sci.* **2019**, *10*, 6174.

[2] H. Hogg, R. G. Wilkins, *J. Chem. Soc.* **1962**, 341.

[3] R. H. Holyer, C. D. Hubbard, S. F. A. Kettle, R. G. Wilkins, *Inorg. Chem.* **1966**, *5*, 622.

[4] X. Xia, Z. Ku, D. Zhou, Y. Zhong, Y. Zhang, Y. Wang, M. J. Huang, J. Tu, H. J. Fan, *Mater. Horizons* **2016**, *3*, 588.

[5] H. Li, L. McRae, C. J. Firby, M. Al-Hussein, A. Y. Elezzabi, *Nano Energy* **2018**, *47*, 130.

[6] G. Cai, X. Wang, M. Cui, P. Darmawan, J. Wang, A. L. S. Eh, P. S. Lee, *Nano Energy* **2015**, *12*, 258.

[7] H. Li, L. McRae, C. J. Firby, A. Y. Elezzabi, *Adv. Mater.* **2019**, *31*, 1.

[8] H. Li, C. J. Firby, A. Y. Elezzabi, *Joule* **2019**, *3*, 2268.

[9] G. Cai, P. Darmawan, X. Cheng, P. S. Lee, *Adv. Energy Mater.* **2017**, *7*, DOI 10.1002/aenm.201602598.

[10] X. Li, Z. Du, Z. Song, B. Li, L. Wu, Q. Liu, H. Zhang, W. Li, *Adv. Funct. Mater.* **2018**, *28*, 1.

[11] G. Cai, P. Cui, W. Shi, S. Morris, S. N. Lou, J. Chen, J. H. Ciou, V. K. Paidi, K. S. Lee, S. Li, P. S. Lee, *Adv. Sci.* **2020**, *7*, 1.

[12] Y. Huang, M. Zhu, Y. Huang, W. Meng, Q. Gong, G. Li, C. Zhi, *J. Mater. Chem. A* **2015**, *3*, 21321.

[13] H. Lv, S. Yang, C. Li, C. Han, Y. Tang, X. Li, W. Wang, H. Li, C. Zhi, *Energy Storage*

Mater. **2021**, *39*, 412.

[14] J. Wang, L. Zhang, L. Yu, Z. Jiao, H. Xie, X. W. Lou, X. Wei Sun, *Nat. Commun.* **2014**, *5*, 1.

[15] X. Chang, R. Hu, S. Sun, J. Liu, Y. Lei, T. Liu, L. Dong, Y. Yin, *Appl. Surf. Sci.* **2018**, *441*, 105.

[16] B. Yang, D. Ma, E. Zheng, J. Wang, *Sol. Energy Mater. Sol. Cells* **2019**, *192*, 1.
